# Determining diagnosis date of diabetes using structured electronic health record (EHR) data: the SEARCH for diabetes in youth study

**DOI:** 10.1186/s12874-021-01394-8

**Published:** 2021-10-10

**Authors:** Kristin M. Lenoir, Lynne E. Wagenknecht, Jasmin Divers, Ramon Casanova, Dana Dabelea, Sharon Saydah, Catherine Pihoker, Angela D. Liese, Debra Standiford, Richard Hamman, Brian J. Wells, the SEARCH for Diabetes in Youth Study Group

**Affiliations:** 1 Department of Biostatistics and Data Science, Division of Public Health Sciences, Wake Forest School of Medicine, Winston-Salem, NC USA; 2 Division of Public Health Sciences, Wake Forest School of Medicine, Winston-Salem, NC USA; 3 Division of Health Services Research, NYU Winthrop Research Institute, NYU Long Island School of Medicine, Mineola, NY USA; 4 Department of Epidemiology, Colorado School of Public Health, University of Colorado Denver, Aurora, CO USA; 5 Division of Diabetes Translation, National Center for Chronic Disease Prevention and Health Promotion, Centers for Disease Control and Prevention, Atlanta, GA USA; 6 Department of Pediatrics, University of Washington, Seattle, WA USA; 7 Department of Epidemiology and Biostatistics, Arnold School of Public Health, University of South Carolina, Columbia, SC USA; 8 Cincinnati Children’s Hospital Medical Center, Cincinnati, OH USA

**Keywords:** Electronic health records, Diabetes mellitus, Algorithms, Infants, Children, Adolescents, Young adults, Epidemiology, Surveillance

## Abstract

**Background:**

Disease surveillance of diabetes among youth has relied mainly upon manual chart review. However, increasingly available structured electronic health record (EHR) data have been shown to yield accurate determinations of diabetes status and type. Validated algorithms to determine date of diabetes diagnosis are lacking. The objective of this work is to validate two EHR-based algorithms to determine date of diagnosis of diabetes.

**Methods:**

A rule-based ICD-10 algorithm identified youth with diabetes from structured EHR data over the period of 2009 through 2017 within three children’s hospitals that participate in the SEARCH for Diabetes in Youth Study: Cincinnati Children’s Hospital, Cincinnati, OH, Seattle Children’s Hospital, Seattle, WA, and Children’s Hospital Colorado, Denver, CO. Previous research and a multidisciplinary team informed the creation of two algorithms based upon structured EHR data to determine date of diagnosis among diabetes cases. An ICD-code algorithm was defined by the year of occurrence of a second ICD-9 or ICD-10 diabetes code. A multiple-criteria algorithm consisted of the year of first occurrence of any of the following: diabetes-related ICD code, elevated glucose, elevated HbA1c, or diabetes medication. We assessed algorithm performance by percent agreement with a gold standard date of diagnosis determined by chart review.

**Results:**

Among 3777 cases, both algorithms demonstrated high agreement with true diagnosis year and differed in classification (*p* = 0.006): 86.5% agreement for the ICD code algorithm and 85.9% agreement for the multiple-criteria algorithm. Agreement was high for both type 1 and type 2 cases for the ICD code algorithm. Performance improved over time.

**Conclusions:**

Year of occurrence of the second ICD diabetes-related code in the EHR yields an accurate diagnosis date within these pediatric hospital systems. This may lead to increased efficiency and sustainability of surveillance methods for incidence of diabetes among youth.

**Supplementary Information:**

The online version contains supplementary material available at 10.1186/s12874-021-01394-8.

## Background

The SEARCH for Diabetes in Youth Study (SEARCH) has documented trends in the incidence and prevalence of diabetes among youth aged less than 20 years in five geographic areas of the United States since 2001 [1–5]. Manual chart review remains the primary method to identify diabetes status, diabetes type, and date of onset, which requires substantial time and effort. Previous work determined that structured electronic health record (EHR) data, which has become widely available, can accurately determine diabetes status and type [6–9]. Given the longitudinal nature of EHR data with associated dates, it is reasonable to consider leveraging the EHR to determine date of diagnosis to enable identification of incident cases.

EHR systems have been increasingly utilized to enhance the efficiency of public health surveillance, particularly in establishing prevalence of disease [10]. However, identifying date of onset of any disease is challenging due to the absence of a structured date field that captures this information. EHR data have been explored as a mechanism to efficiently identify incident cases of diabetes. Studies often define this incident population through a requirement that a person is present in the EHR network and free from evidence of diabetes for a defined length of time or for a number of prior outpatient visits [11–14]. First evidence defined by a well-reasoned EHR algorithm is then considered date of onset. Pantalone characterized newly diagnosed type 2 diabetes cases among adults as those with at least two primary care outpatient visits prior to the first appearance of a diabetes-related ICD code [11]. Date of diagnosis was determined by time of anti-hyperglycemic medication, ICD-9 code, or laboratory value consistent with diabetes. Another study identified incident prediabetes cases by elevated glycated hemoglobin (HbA1c) and glucose values occurring after at least 2 years of longitudinal EHR data without evidence of diabetes or prediabetes (qualifying ICD diabetes code, glucose, HbA1c, or metformin prescription) [15]. The scope of previous work is largely focused on the identification of incident cases for observation or follow-up care, in which case precise date of diagnosis is not essential. Limitations include the lack of validation of algorithms [15] or an extremely small sample size for validation (*n* = 20) [11].

Therefore, the purpose of the present study is to adapt previous research to surveillance efforts and to validate EHR-based algorithms to determine date of diagnosis in a large sample of youth with diabetes. The SEARCH for Diabetes in Youth study provides an ideal opportunity to evaluate this question as EHR-based methods can be compared to a chart-reviewed gold standard to comprehensively fulfill the needs of automated case registration and increase surveillance efficiency.

## Methods

### Search

The SEARCH for Diabetes in Youth study has conducted population-based incidence and prevalence ascertainment of non-gestational diabetes in youth since 2001 [1–5]. SEARCH identifies youth diagnosed under the age of 20 years at the following locations: health plan members in seven counties in Southern California, the state of Colorado, Native American reservations in Arizona and New Mexico, eight counties in Ohio, the state of South Carolina, and five counties in Washington. Cases are also identified by a variety of sources that include referrals from physicians and other health care providers, community health systems, and diabetes registries. In SEARCH, a diabetes case is determined by physician diagnosis. This determination can be made by provider report, medical record review, or self-report.

Three hospital systems that are part of the SEARCH case ascertainment network participated in this study: Cincinnati Children’s Hospital, Cincinnati, OH, Seattle Children’s Hospital, Seattle, WA, and Children’s Hospital Colorado, Denver, CO. The study was approved by the SEARCH coordinating center (Wake Forest University Health Sciences Institutional Review Board; IRB00015926) with waivers of informed consent and Health Insurance Portability and Accountability Act authorization. This study was also approved by the local Institutional Review Boards of the participating sites. Methods were carried out in accordance with the Declaration of Helsinki and all other relevant guidelines and regulations**.** Two of these study sites use EHRs developed by Epic (Verona, WI) while the other site employs an EHR developed by Cerner EHR (Kansas City, MO).

### Case identification

This work originates from a project designed to explore detection of diabetes status, diabetes type, and date of diagnosis within a cohort of possible 2017 prevalent cases. All potential cases of youth with diabetes aged less than 20 years in 2017 were extracted from the EHR at three hospital systems through the use of a highly sensitive algorithm. The sensitive algorithm included at least one inpatient or outpatient clinical encounter in 2017 and at least one of the following criteria: a diabetes-related International Classification of Disease, 10th Revision, (ICD-10) diagnosis code, a glycated hemoglobin A1c ≥ 6.5%, a fasting or random glucose value ≥126 mg/dl and 200 mg/dl respectively, or a diabetes-related medication [16].

### Gold standard for date of diagnosis

Potential diabetes cases were matched to the SEARCH registry, which included date of diagnosis and diabetes type from previous medical record review. Diabetes cases identified by the sensitive algorithm that were not already in the registry underwent the same review process. Calendar month and year of date of diabetes diagnosis were recorded for each subject and used as the gold standard to which date of diagnosis algorithms were compared.

### Electronic health record data

Structured outpatient and inpatient EHR data were extracted for patients within the following domains: demographics, laboratory measurements, diagnosis codes, medications, and vital signs. Dates were recorded as calendar month and year. Each site removed protected health information prior to transmitting the data to the coordinating center for harmonization and analysis.

### Diabetes status and type

Previous research in SEARCH demonstrated that structured EHR data yields respectable metrics for determining diabetes status and type [6–8, 16]. The presence of at least two ICD-10 codes (E08-E13.x, P70.2, O24.0x, and O24.1x) determines status well, and a preponderance of type 1 diabetes, type 2 diabetes, and other diabetes (non-type 1 or type 2) codes can accurately determine type when paired with limited manual chart review of type 2 diabetes and other type cases [9]. Given the excellent metrics in determining diabetes status and the similarity to other algorithms in the literature, the authors applied this rule-based ICD-10 approach to all accumulated diagnosis data from the point of EHR entry through 12/31/2017 to identify probable diabetes cases. We tested date of diagnosis algorithms within this population.

### Inclusion criteria

Probable cases according to the rule-based ICD-10 algorithm were included in the analysis. Eligibility criteria were intended to mimic a real-world application in which one would not know true diabetes status or date of diagnosis and would therefore be unable to subset the population according to either of these parameters. Patients were restricted to those first detected in the EHR from 1/1/2009 through 12/31/2017 as EHR systems were limited prior to 2009. This reduced the number of cases with incomplete data at the time of diagnosis. We considered limiting cases to those with a pre-defined period of time in the EHR without evidence of diabetes. We did not pursue this approach as this would have substantially reduced the size of the analytic cohort, and we found that performance metrics remained strong without the additional requirement.

### Date of diagnosis algorithms

We considered two algorithms to determine date of diagnosis: an ICD code algorithm and a multiple-criteria algorithm. These algorithms were developed by a multidisciplinary team of clinicians, epidemiologists, and informaticians who participated in SEARCH and have extensive experience with childhood diabetes. The ICD code algorithm was defined as the time of occurrence of second diabetes diagnosis code (ICD-9: 249–250.x, 357.2, 362.0x, 366.41, 648.0x, 775.1 and/or ICD-10: E08-E13.x, P70.2, O24.0x, O24.1x) and was based upon previous success in the identification of prevalent diabetes cases [9]. Both ICD-9 and ICD-10 codes were utilized as the span of potential diagnosis dates preceded the implementation of ICD-10 in October of 2015. The multiple-criteria algorithm was defined as the time of occurrence of the first diabetes-related diagnosis code, or elevated glycated hemoglobin ≥6.5%, or elevated glucose (≥ 126 mg/dl fasting, ≥ 200 mg/dl random), or diabetes-related medication (Alpha Glucosidase Inhibitors, Dipeptidyl Peptidase-4 (DPP4) Inhibitors, Glucagon-like Protein-1 (GLP-1) Receptor Agonists, Insulin, Meglitinides, Sodium-glucose co-transporter-2 (SGLT2) inhibitors, Sulfonylureas, Thiazolidinediones, and other medications identified by clinicians). This combination of variables was based upon strong association with diabetes status, presence in the literature [6, 8, 11, 14, 15], and adequate data availability in the EHR.

### Statistical methods

All analyses were conducted using R version 3.6.2 (R foundation for Statistical Computing). We assessed the performance of the rule-based ICD-10 status algorithm for diabetes status with accuracy, sensitivity, and specificity. Performance between each date of diagnosis algorithm compared to the gold standard calendar year of diagnosis was quantified by percent agreement (number of observations where predicted calendar year matched the gold standard year divided by the total number of probable diabetes cases identified by the rule-based ICD-10 status algorithm) and Cohen’s Kappa for interrater reliability [17]. McNemar’s test identified if the marginal proportions between algorithms differed overall and within each diabetes type. A two proportion z-test identified differences in proportions correctly classified between type 1 and type 2 diabetes cases within each algorithm. We deemed results of all tests statistically significant at *P* < 0.05. We examined concordance between predicted and gold standard calendar month and year for the ICD code algorithm by scatterplot with underlying distribution by gold standard diagnosis year. We inspected performance over time visually by line graph with 95% confidence intervals for each year of diagnosis. Visualizations were limited to 2009–2017 due to lack of EHR data prior to 2009; type 2 cases were limited to 2012–2017 due to the small number of cases (*n* = 12) with gold standard date from 2009 to 2011. While year alone is most relevant for surveillance purposes, month and year of diagnosis is important for a variety of other reasons beyond the scope of this paper. Therefore, we also report overall percent agreement, Kappa, and McNemar’s test for the algorithms compared to the gold standard calendar month and year (plus or minus 1 month) and visually examined performance over time.

## Results

Among potential cases (*n* = 6386), the rule-based ICD-10 algorithm identified 3777 probable cases of diabetes. Table 1 displays the characteristics of these cases by site. Cases where SEARCH staff could not determine a diagnosis date from the medical record (*n* = 19) or who were ineligible according to SEARCH criteria (*n* = 46, e.g., geographic status, institutionalization, etc.) were excluded. Many cases (*n* = 2331, 61.7%) first appeared with some EHR-based evidence of diabetes during the same month they first appeared in the EHR network and 1916 (50.7%) had a concordant gold standard month and year of diagnosis at this time (Supplemental Table 1).

**Table 1:** Characteristics of youth identified by rule-based ICD-10 algorithm by SEARCH site

Variable	Site A	Site B	Site C	Total
	*n* = 1217	*n* = 1305	*n* = 1255	*n* = 3777
**Age group in 2017 (years), n (%)**				
0–4	50 (4.1)	50 (3.8)	93 (7.4)	193 (5.1)
5–9	194 (15.9)	259 (19.8)	348 (27.7)	801 (21.2)
10–14	459 (37.7)	438 (33.6)	453 (36.1)	1350 (35.7)
15–19	514 (42.2)	558 (42.8)	361 (28.8)	1433 (37.9)
**Mean age of diagnosis in years (SD)**	8.9 (4.3)	8.7 (4.3)	7.3 (4.3)	8.3 (4.4)
**Sex, n (%)**				
Female	596 (49.0)	598 (45.8)	623 (49.6)	1817 (48.1)
Male	621 (51.0)	707 (54.2)	632 (50.4)	1960 (51.9)
**Race, n (%)**				
White	959 (78.8)	835 (64.0)	862 (68.7)	2656 (70.3)
Black	187 (15.4)	109 (8.4)	67 (5.3)	363 (9.6)
Other/Unknown	71 (5.8)	361 (27.7)	326 (26.0)	758 (20.1)
**Ethnicity, n (%)**				
Hispanic	47 (3.9)	139 (10.7)	228 (18.2)	414 (11.0)
Non-Hispanic or unknown	1170 (96.1)	1166 (89.3)	1027 (81.8)	3363 (89.0)
**SEARCH diabetes type, n (%)**				
Type 1	1009 (88.4)	1166 (90.3)	1165 (94.2)	3340 (91.0)
Type 2	115 (10.1)	110 (8.5)	52 (4.2)	277 (7.5)
Other (non-type 1 or type 2)	18 (1.6)	15 (1.2)	20 (1.6)	53 (1.4)

Values are presented as mean (standard deviation) for continuous variables and count (%) for categorical variables

The rule-based ICD-10 algorithm for identification of diabetes performed well with an overall accuracy of 0.98, sensitivity of 0.99, and a specificity of 0.96. Of those considered to be probable diabetes cases, 94.1% were correctly classified by the rule-based ICD-10 approach for diabetes type. A detailed classification matrix is included as Supplemental Figure 1.

Within probable diabetes cases, the ICD code algorithm demonstrated 86.5% (3267/3777) agreement with the gold standard year of diagnosis and a high interrater reliability (Kappa = 0.85). This algorithm was not equivalent in case classification to the multiple criteria algorithm (*p* = 0.006), which demonstrated 85.9% (3246/3777) agreement (Kappa = 0.84). The ICD code algorithm correctly classified 40 diabetes cases that the multiple-criteria algorithm misclassified (incorrectly predicted the year of diagnosis), and the multiple-criteria algorithm correctly classified 19 that the ICD code algorithm misclassified. When compared to gold standard calendar month and year (plus or minus 1ne month), the ICD code algorithm demonstrated 85.1% (3215/3777) agreement (Kappa = 0.83) and statistically differed in classification compared to the multiple-criteria algorithm (*p* = 0.002), which demonstrated 84.4% (3189/3777) agreement (Kappa = 0.82). The denominator for these calculations included 107 false positives for which the predicted date of diagnosis could not be compared to a gold standard date of diagnosis as these were not true/validated cases of diabetes. Cases with a diagnosis date prior to 2009 (*n* = 119) were also incorrectly classified due to lack of EHR data. These remained in the denominator as systematic exclusion is not possible in future application.

**Fig. 1:**
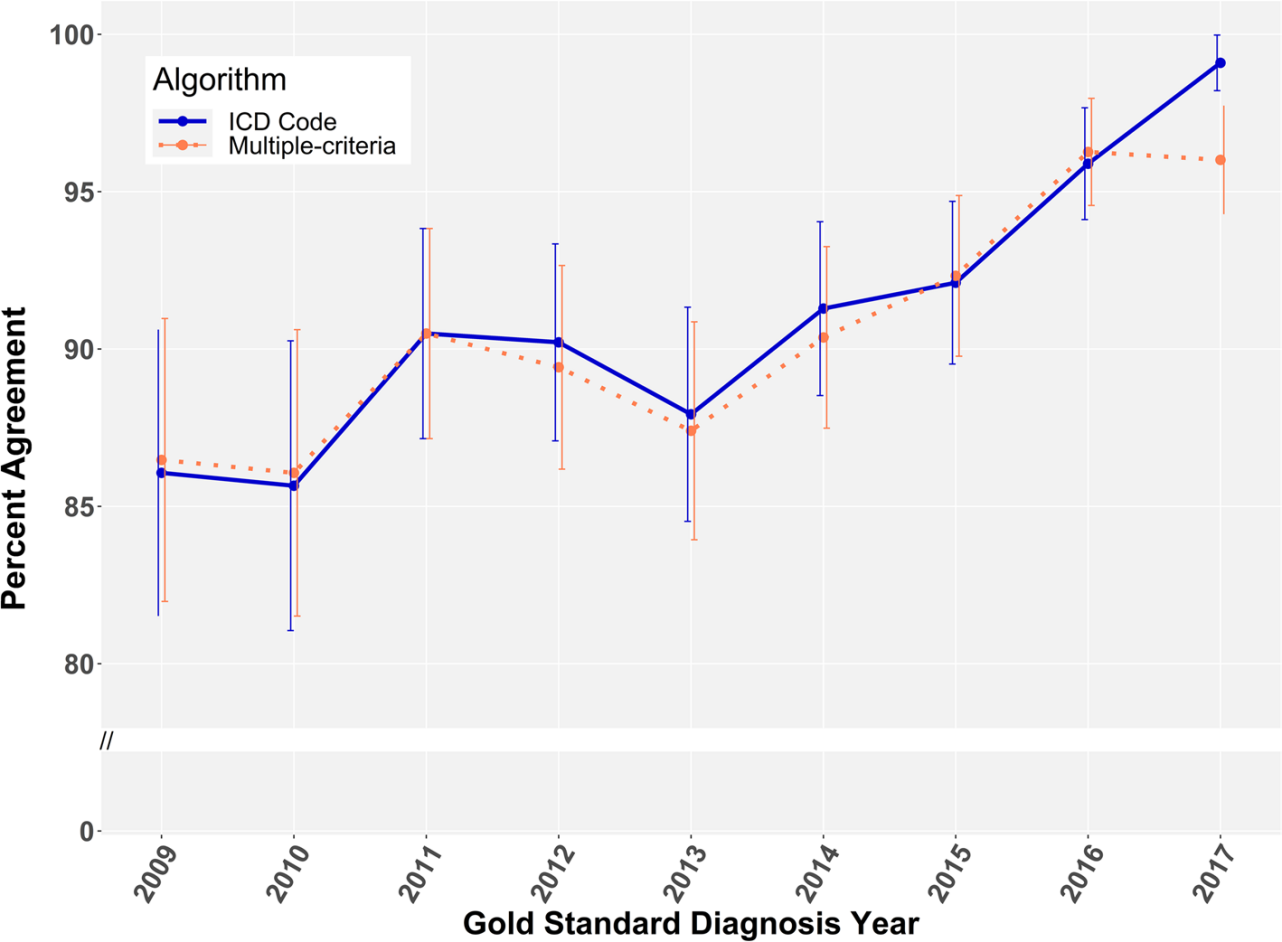
Algorithm percent agreement with gold standard year of diagnosis (2009–2017). Non-diabetes observations (*n* = 107) incorrectly identified by the rule-based ICD-10 algorithm and diabetes cases (*n* = 119) with gold standard date of diagnosis preceding 2009 are not visualized

Percent agreement by calendar year improved over time from 2009 through 2017 and exceeded 95% for 2016 and 2017 (Fig. 1). Agreement differed modestly across sites and was initially lower at site C, but improved and consistently remained greater than 90% for 2016 and 2017 at all sites (Supplemental Figure 2). Percent agreement within calendar month also improved over time and exceeded 95% in 2016 and 2017 for the ICD code algorithm (Supplemental Figure 3).

**Fig. 2:**
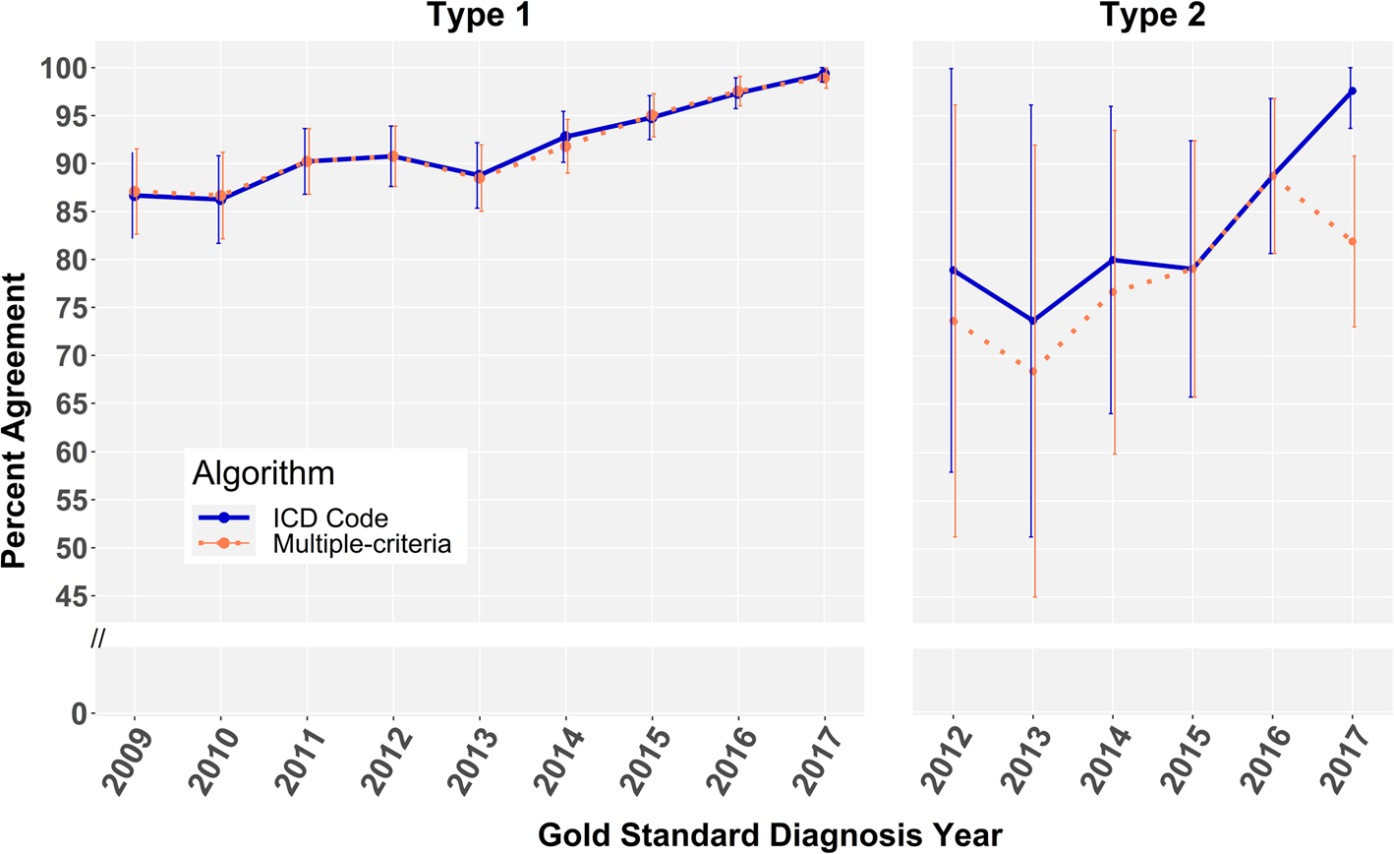
Algorithm percent agreement with true gold standard year of diagnosis by Type 1 and Type 2 diabetes. Type 2 cases limited to 2012–2017 due to small number of cases with date of diagnosis from 2009 to 2012 (*n* = 12)

Figure 2 displays percent agreement by gold standard year of diagnosis for type 1 diabetes (2009–2017) and type 2 diabetes cases (2012–2017). The ICD code algorithm and multiple-criteria date of diagnosis algorithms made considerably different predictions for type 2 cases (*p* = 0.010), but not for type 1 (*p* = 0.439) or other type cases (*p* = 0.527). The ICD code algorithm correctly classified diagnosis year in 89.6% (2991/3340) of type 1 diabetes cases and 86.6% (240/277) of type 2 diabetes and showed no statistical difference between proportions correctly classified by year (*p* = 0.132). The multiple-criteria algorithm classified diagnosis year correctly in 89.5% (2988/3340) of type 1 diabetes cases and 80.9% (224/277) of type 2 diabetes cases and statistically differed by type (*p* < 0.001). These trends persist upon examination of agreement with calendar month and year (Supplemental Figure 4). Percent agreement for other types of diabetes were 67.9% (36/53) and 64.2% (34/53) for the ICD code and multiple-criteria algorithms, respectively.

**Fig. 3:**
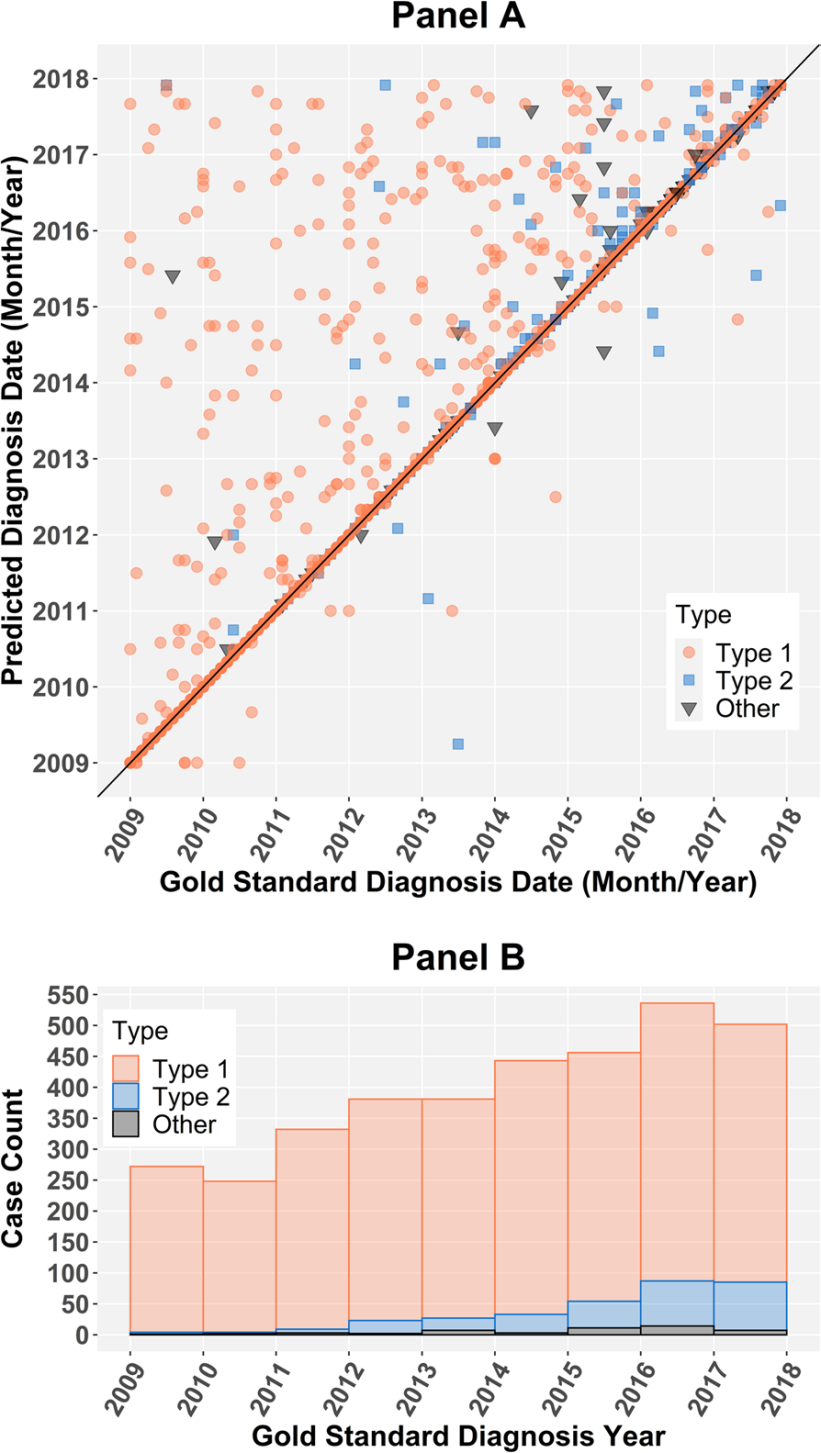
ICD code algorithm and gold standard date of diagnosis concordance and accompanying distribution. In Panel **A**, the diagonal line represents perfect alignment of calendar month/year between the predicted and gold standard date of diagnosis for diabetes cases. The accompanying histogram in Panel **B** demonstrates frequency of diabetes cases by type and within each year as the intensity of the 45 degree line is not easily discernable

Figure 3 further illustrates the accuracy of the ICD code algorithm for predicting date of diagnosis. The diagonal line represents perfect agreement between predicted and gold standard month and year. When dates disagreed, the true date of diagnosis was often earlier than the predicted date. While this trend was also present for the multiple-criteria algorithm, visual inspection showed a larger number of cases with a true date of diagnosis subsequent to the predicted date (Supplemental Figure 5).

## Discussion

We demonstrate that structured EHR data can accurately identify the year of diagnosis in approximately 86% of youth with diabetes. Of the two algorithms we evaluated, we recommend using the ICD code algorithm (year of occurrence of the second ICD-9 or ICD-10 diabetes code) to identify onset of diabetes in practice. First, the ICD code algorithm performed better at classification of cases overall and type 2 diabetes cases against the gold standard when compared to the multiple-criteria algorithm. Second, performance was high and did not differ between type 1 diabetes (89.6%) and type 2 diabetes (86.6%) cases. Third, the utilization of diagnosis codes is simple and avoids the need to harmonize and process additional structured data elements in the EHR. Finally, this approach is consistent with our previous work for determining diabetes status and type in youth, and facilitates an easier combination of methods to comprehensively determine diabetes status, type, and date of diagnosis.

We observed improvement in the accuracy of the algorithms over the period from 2009 through 2017. This trend persisted for all age groups at time of diagnosis (0–4, 5–9, 10–14, and 15–19 years of age) and did not appear to be an artifact of the cohort definition that restricted the population to those less than 20 years of age at the end of 2017. Additionally, type 2 cases which are typically diagnosed later in childhood were thus observed in higher proportions from 2012 to 2017. This only bolsters our confidence in temporal improvement as these cases showed slightly lower agreement compared to type 1 cases although not statistically so for the ICD code algorithm. Although there was no underlying trend or impact in the number of cases diagnosed prior to EHR entry over time, the proportion of cases that entered the EHR at the time of diagnosis decreased each year for all age groups. We are unsure why this is the case, but this is yet another indicator of improvement over time that may be affected by dynamic EHR structures and clinical practices. Temporal improvement may be due to an increase in the amount of available structured data as EHR adoption extends to additional internal departments or as networks acquire new clinical practices over time. Systematic improvement in coding procedures may also contribute. For example, October 2015 marked the implementation of ICD-10, and new coding introduced enhancements to classification of diabetes type and spurred further educational coding initiatives for healthcare personnel. Whatever the underlying reason, the improvement in performance observed over time has important implications for future surveillance activities and suggests that performance accuracy may exceed 90% when this algorithm is applied prospectively.

Our results are likely a conservative estimate of the accuracy of the algorithm in predicting date of diagnosis. The denominator of our calculation is inflated with probable cases whose gold standard diagnosis dates are between 1998 and 2008, the period preceding the time that sites in this study had well-established EHR systems. Identifying a retrospective diagnosis date in the era of paper-based records is notably challenging and we attempted to limit the number of these cases by requiring a person’s first instance of any EHR data to occur after 2008. This issue will be lessened in future studies as length of EHR-established time increases. The denominator is also inflated by 107 non-cases incorrectly identified as probable diabetes cases by the rule-based ICD-10 status algorithm. Removing these misclassified cases increases percent accuracy to 89.0% for the ICD code algorithm and 88.4% for the multiple-criteria algorithm. The algorithms perform well despite these limitations.

For the ICD code algorithm for date of diagnosis, performance trended towards better classification of type 1 cases compared to type 2. This is likely due to differences in the presentation of disease as very acute in type 1 diabetes but prolonged in type 2 diabetes. There are ways to improve performance. We have previously recommended that a targeted chart review be employed in the small number of type 2 diabetes and other type cases (*n* = 465, 12.3% of total cases) to manually identify type of diabetes; such review could also be used to determine date of diagnosis, which would improve classification and percent agreement [9]. This method prioritizes efficiency through automation as the primary method of ascertainment with modest augmentation by chart review. Another option is scouring free text and physician notes through natural language processing in order to supplement structured data, although one must weigh the resources required to adapt an algorithm with the amount of improvement one can achieve [18, 19].

We explored a number of variations in algorithms in order to select the most efficient algorithms presented in this paper. The first diabetes-related ICD-9 or ICD-10 code for determining date of diagnosis was considered, but this algorithm classified fewer cases correctly with respect to precise gold standard calendar month and year and was not statistically equivalent in performance to the date of two or more ICD codes (*p* < 0.001). This in conjunction with previous research [9] served as a basis for proceeding with two or more cumulative ICD codes for our analyses. We speculate that the date of the first diagnosis code represents and exploration of possible diabetes by a clinician, while a second code serves as a confirmation of diagnosis and thus agrees more closely with the gold standard. Furthermore, using two codes aligns the algorithm for date of diagnosis with confirmation of diabetes status by the rule-based ICD-10 status algorithm [9, 16]. We chose not to analyze single-variable algorithms such as glucose level or diabetes medications due to challenges in missing data and outliers; hence, we selected a multiple-criteria algorithm. Variables were not limited or stratified based on inpatient or outpatient class designation as this was largely incomplete. Using these variables and designations would have resulted in a large proportion of cases with a missing predicted diagnosis date.

Evaluation of electronic algorithms for diabetes surveillance are limited by the lack of a gold standard. We were able to overcome this limitation in SEARCH with our large annotated dataset of diabetes cases in youth. Other strengths include the three study sites in different regions of the country, two different EHR systems, and the large number of type 2 diabetes cases (a condition less prevalent in youth).

These algorithms are applicable to a population aged less than 20 years and future research should test applicability within adult populations. There are differences in epidemiologic patterns by age such as a higher incidence of type 2 diabetes for adults compared to their younger counterparts. While this study demonstrated good performance among type 2 cases, other factors that may affect performance such as length of time an EHR has been established are unknown and beyond the scope of this study.

A limitation is our inability to test the algorithms outside of these three pediatric healthcare systems. We hypothesize that accuracy may differ across systems and sites due to underlying institutional coding practices, geographic patient mobility in and out of network, and presence of competing local clinical healthcare institutions that could affect completeness of one’s EHR record at the time of diagnosis within a single network. Augmentation with claims data or integration of data from external EHR’s in close geographic proximity could help distinguish new cases especially among those who show evidence of diabetes upon EHR network entry. Ideally, an integration of all EHRs that serve a geographic region could provide comprehensive information regarding care over time and could provide a robust examination of date of diagnosis algorithms.

## Conclusions

In conclusion, the date of occurrence of the second ICD-9 or ICD-10 diagnosis code demonstrated good performance for the estimation of year of diagnosis for youth with type 1 diabetes and type 2 diabetes, and showed improvement over time within these pediatric healthcare systems. Algorithms derived from structured EHR data may increase the efficiency of childhood diabetes surveillance efforts when compared to more resource-intensive methods to determine date of diagnosis.

## Supplementary Information

**Additional file 1.**

## Data Availability

The deidentified datasets analyzed for the current study will be made available upon reasonable request to the corresponding author, with permission from the study principal investigator, and upon completion of a data use agreement. The data use agreement will require that the data be used only for research purposes, that no attempts will be made to identify individual participants, that the data will be kept secure, that the user will not distribute the data to other researchers, that the user will return the files or destroy them once the project is completed, and that the user will acknowledge the data source.

## Abbreviations

EHR: Electronic health record
ICD: International classification of diseases
