# Supplementary material for: Determining diagnosis date of diabetes using structured electronic health record (EHR) data: the SEARCH for diabetes in youth study
Source: BMC Med Res Methodol. 2021 Oct 10;21:210. doi: 10.1186/s12874-021-01394-8 (PMC8502379; doi:10.1186/s12874-021-01394-8)
Supplement: Supplementary file 1 — Additional file 1. [file 12874_2021_1394_MOESM1_ESM.zip › Supplemental Table 1.docx]

**Supplemental Table 1.** Time in EHR for diabetes cases identified by the rule-based ICD-10 status and type algorithm.

| Rule-based ICD-10 Algorithm Diabetes Type | **Type 1** | **Type 2** | **Other** | **Total** |
| --- | --- | --- | --- | --- |
| Case Count | 3314 | 419 | 44 | 3777 |
| Mean total months in EHR  (SD) | 60.5 (30.3) | 57.2 (32.6) | 51.2 (32.5) | 60.0 (30.6) |
| Diagnosis calendar month/year is same as EHR entry  n (%) | 1799 (54.3) | 111 (26.5) | 6 (13.6) | 1916 (50.7) |
| **Evidence: Time occurrence of two or more ICD diabetes codes** | | | | |
| Mean months without evidence in months (SD) | 14.1 (24.6) | 32.70 (32.13) | 26.11 (28.19) | 16.24 (26.22) |
| Enter EHR and evidence within the same month  n (%) | 2114 (63.8) | 133 (31.7) | 8 (18.2) | 2255 (59.7) |
| Entry into EHR and first diabetes evidence within 6 months  n (%) | 2258 (68.1) | 161 (38.4) | 16 (36.4) | 2435 (64.5) |
| Entry into EHR and first diabetes evidence within 12 months  n (%) | 2357 (71.1) | 177 (42.2) | 18 (40.9) | 2552 (67.6) |
| **Evidence: First occurrence of any item in the multiple-criteria algorithm** | | | | |
| Mean months without evidence in months (SD) | 13.7 (24.4) | 26.6 (29.9) | 18.89 (23.0) | 15.2 (25.4) |
| Enter EHR and evidence within the same month  n (%) | 2165 (65.3) | 152 (36.3) | 14 (31.8) | 2331 (61.7) |
| Entry into EHR and first diabetes evidence within 6 months  n (%) | 2281 (68.8) | 178 (42.5) | 20 (45.5) | 2479 (65.6) |
| Entry into EHR and first diabetes evidence within 12 months  n (%) | 2379 (71.8) | 195 (46.5) | 23 (52.3) | 2597 (68.8) |

Start of time in the EHR was considered the first month and year in which a patient had any recorded EHR measurement within any domain (diagnosis codes, vitals, laboratory measurements, and medications.)
