# Supplementary figures and images for: Determining diagnosis date of diabetes using structured electronic health record (EHR) data: the SEARCH for diabetes in youth study
Source: BMC Med Res Methodol. 2021 Oct 10;21:210. doi: 10.1186/s12874-021-01394-8 (PMC8502379; doi:10.1186/s12874-021-01394-8)

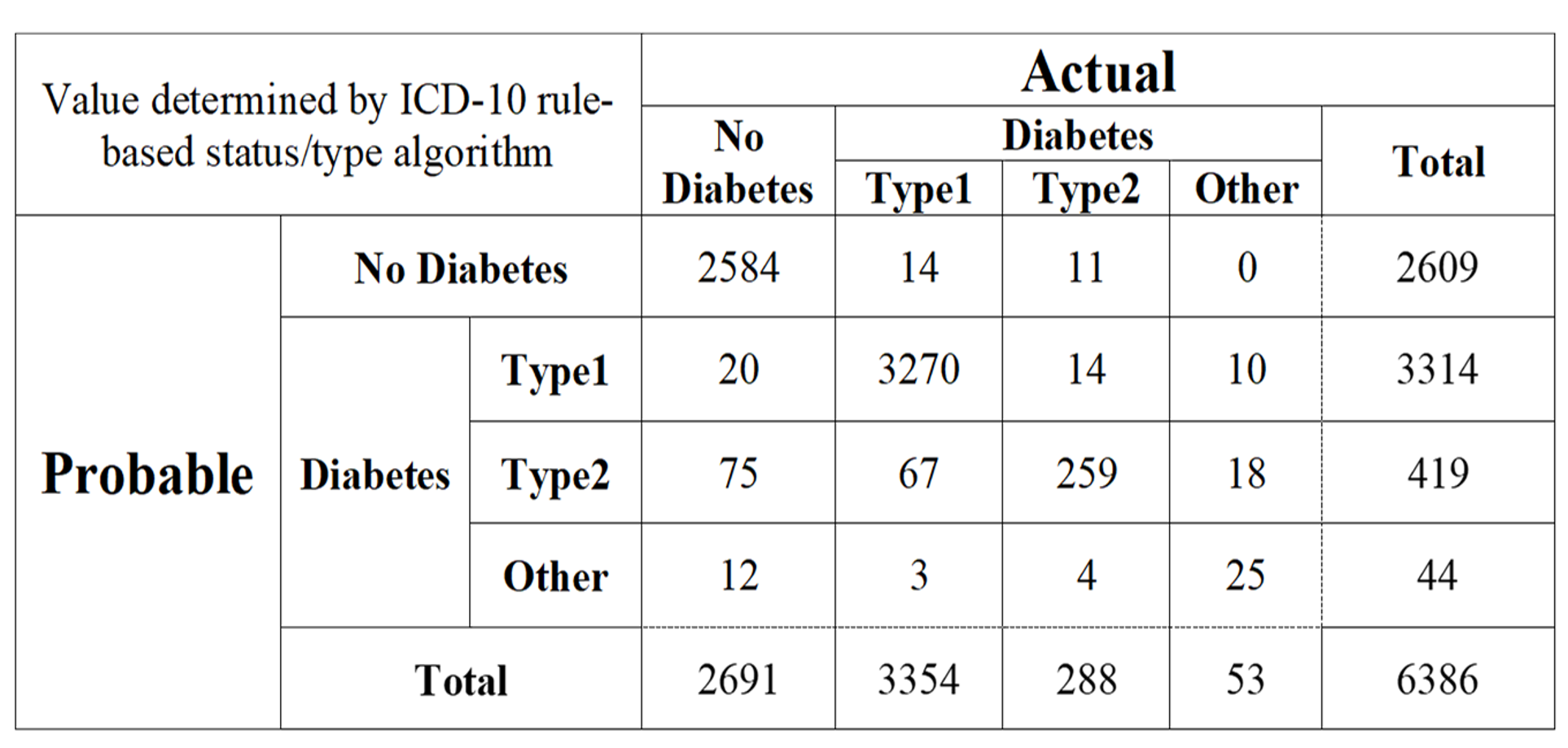

Supplement: Supplementary file 1 — Additional file 1. [file 12874_2021_1394_MOESM1_ESM.zip › supplemental figure 1.png]

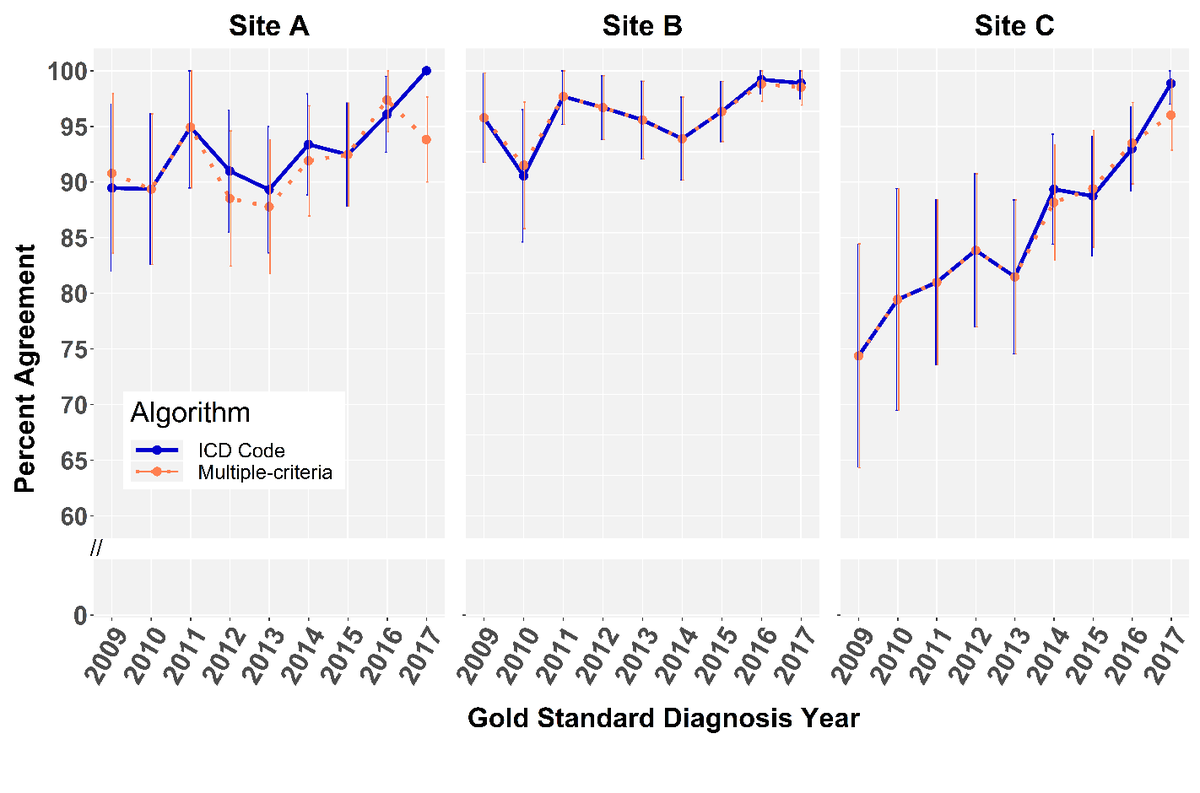

Supplement: Supplementary file 1 — Additional file 1. [file 12874_2021_1394_MOESM1_ESM.zip › Supplemental Figure 2.png]

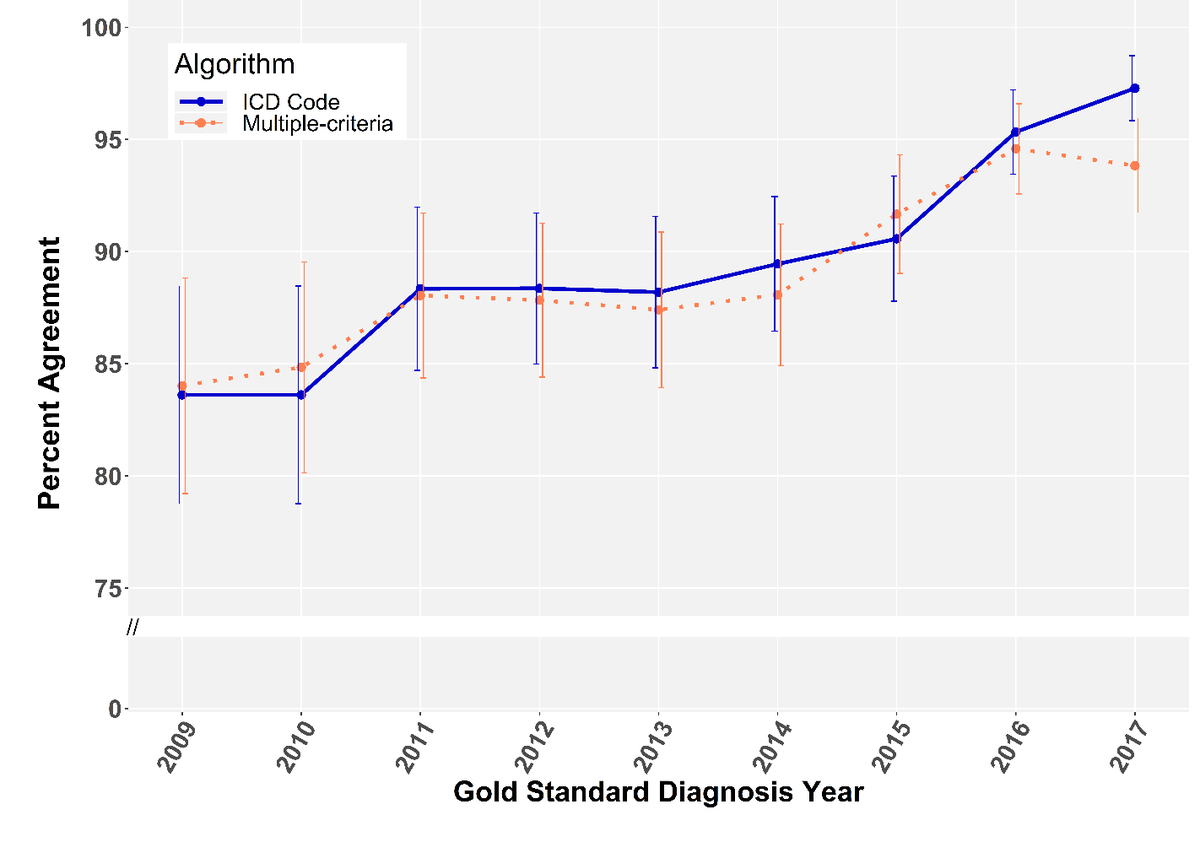

Supplement: Supplementary file 1 — Additional file 1. [file 12874_2021_1394_MOESM1_ESM.zip › Supplemental Figure 3.png]

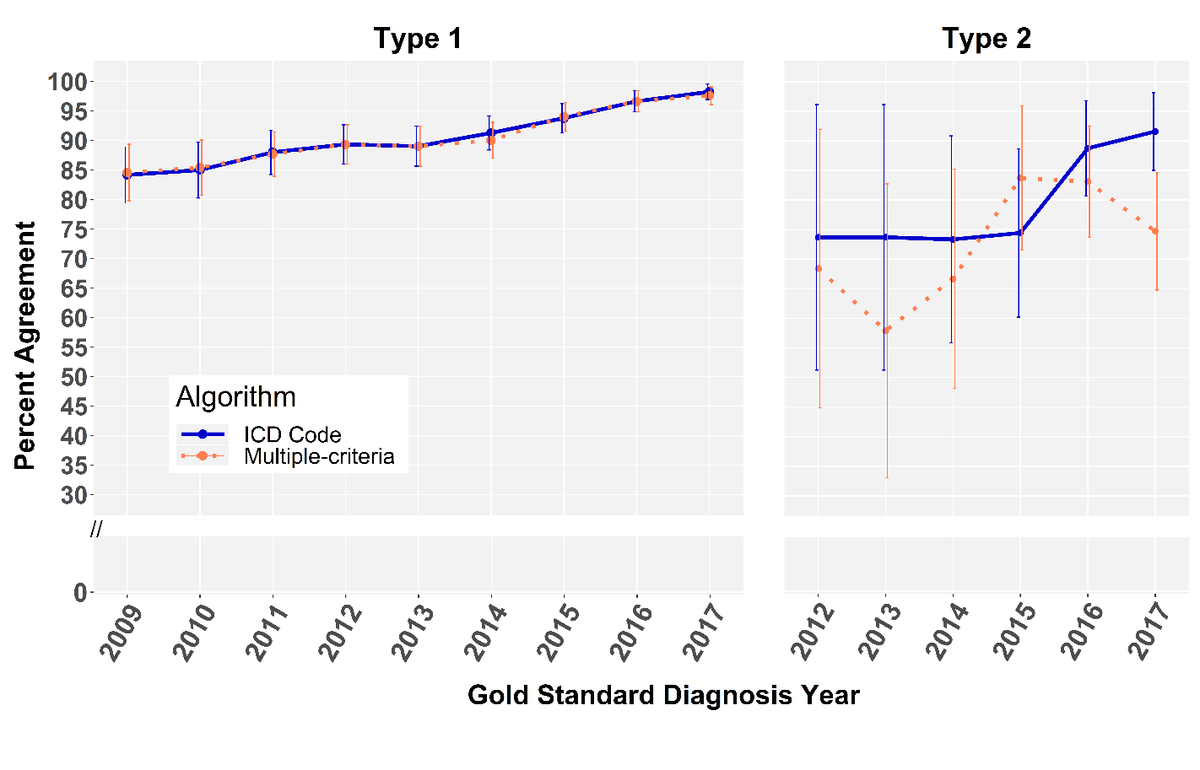

Supplement: Supplementary file 1 — Additional file 1. [file 12874_2021_1394_MOESM1_ESM.zip › Supplemental Figure 4.png]

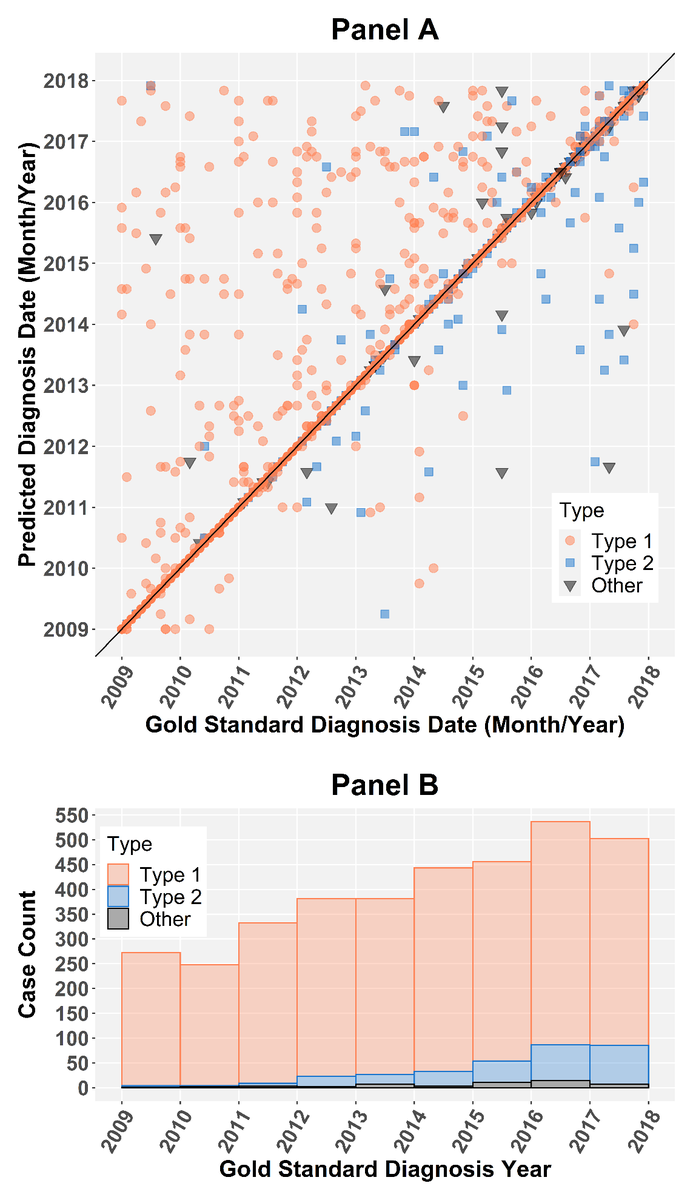

Supplement: Supplementary file 1 — Additional file 1. [file 12874_2021_1394_MOESM1_ESM.zip › Supplemental Figure 5.png]
